# Heel riser height and slope gradient influence the physiology of ski mountaineering—A laboratory study

**DOI:** 10.3389/fphys.2023.1159728

**Published:** 2023-04-19

**Authors:** Michael Lasshofer, John Seifert, Anna-Maria Wörndle, Thomas Stöggl

**Affiliations:** ^1^ Department of Sport and Exercise Science, University of Salzburg, Salzburg, Austria; ^2^ Department of Health and Human Development, Montana State University, Bozeman, MT, United States; ^3^ Red Bull Athlete Performance Center, Salzburg, Austria

**Keywords:** human-equipment interaction, sports equipment, winter sports, olympic sport, performance testing

## Abstract

In ski mountaineering it is the goal to reach the top of a mountain by sheer muscle force. The specific equipment (flexible boot, only toe fixated binding, and a skin on the ski to prevent from slipping backwards) enables the skier to move up the hill ergonomically, where the heel part of the binding offers a special adaptation possibility. The so-called riser height supports the heel standing height and can be adjusted to individually preferred settings. General recommendations suggest using lower heel support in flat ascents and higher heel support in steep ascents to maintain upright posture and lower the strain. Still, it remains unclear whether the application of riser height affects the physiological response during ski mountaineering. This study was designed to investigate the effects of riser height on physiological response during indoor ski mountaineering. Nineteen participants took part in the study and walked on a treadmill with ski mountaineering equipment. The three available riser heights (low, medium, and high) were applied randomized at 8%, 16%, and 24% gradient. Results show that global physiological measurements like heart rate (*p* = 0.34), oxygen uptake (*p* = 0.26) or blood lactate (*p* = 0.38) values were not affected by changes in riser height. But local measurements of muscle oxygen saturation were affected by the riser height. Additionally comfort and rating of perceived exertion were also prone to changes in riser height. These results suggest differences on local measurements and perceived parameters, while global physiological measurements did not change. The results are in line with the existing recommendations but need to be confirmed in an outdoor setting as well.

## Introduction

Ski mountaineering (skimo) is a multi-faceted winter sport where equipment and environment play key roles in performance (Bortolan et al., 2021). Like in many other sports, skimo exists primarily in two domains, as a recreational activity and as a competitive sport. While racing strives for optimizing performance combined with light equipment (Bortolan et al., 2021), as a recreational sport, skimo combines alpine and Nordic skiing characteristics and provides the chance for skiers to enjoy the outdoors in a unique and adventurous way. The physiological strain of skimo can be high, and racing was described as one of the most strenuous endurance sports (Duc et al., 2011; Praz et al., 2014; Lasshofer et al., 2021; Kayser and Mariani, 2022), with high relevance of performance testing and analysis (Menz et al., 2021; Schöffl et al., 2022; Zimmermann et al., 2022).

Crucial to the success of a ski tour is not only the planning of the tour and the fitness level, but also the equipment used. For example, heavier equipment can increase the energy cost by 1.7% for each percent of bodyweight added to the ankles (Tosi et al., 2009). This added weight has a significant impact on skimo racing performance (Bortolan et al., 2021), but appears to be negligible in recreational skimo tours. A wide range of skis, bindings, and boots are available. However, all boot and binding systems have in common a walking and a skiing mode. While the skiing mode compares well with alpine skiing systems, the walking mode clearly differs. Within the walking mode, the heel is not connected to the binding and the tip of the boot pivots. Pivoting and a flexible boot cuff allow for walking by an increase in lower limb joints range of motion, compared to the skiing mode. As a very specific part, the rear part of the binding offers an adjustable heel support. This so-called riser height makes it possible to alter the height of the heel support while climbing according to individual preference. General recommendations suggest using a higher riser height at steeper slope gradients and lower riser heights at flatter slope gradients to maintain an upright posture and reduce calf muscle strain (Vives, 1999; Winter 2001; House et al., 2019). Biomechanical analysis of the riser height showed a larger range of motion in lower limb joints by using a lower riser height, accompanied by a lower step frequency, but greater step length. Mechanical efficiency of skimo was not influenced by the application of different riser heights (Lasshofer et al., 2022).

Nevertheless, it remains unclear whether these recommendations are tenable concerning physiological variables and if so, like suggested by Sunde et al. (2021), certain slope gradients can be linked to the application of riser heights. Therefore, this study investigated the effect of riser heights on physiological variables and subjectively emphasized variables during treadmill skimo. We hypothesize that the application of higher riser heights at steeper slope gradients and lower riser heights at flatter slope gradients have a benefit on global physiological variables (heart rate, blood lactate, and oxygen consumption), local physiological variables (muscle oxygen saturation and electromyography signal), perceived exertion (Borg scale), and perceived comfort.

## Materials and methods

### Participants

Participants were recruited by a public invitation and had to be between 18 and 50 years old and practice skimo regularly during the winter season, but do not participate in skimo races. Only male participants were included due to the availability of male specific equipment. Nineteen individuals who matched these criteria participated in the study. Anthropometric data and habitual training load are presented in Table 1. All participants volunteered and gave written informed consent. The study was approved by the ethical committee of the University of Salzburg (EK-GZ: 36/2018).

**TABLE 1:** Age, anthropometrics, equipment and training (*n* = 19).

	Mean ± SD	Min	Max
Age [yr]	34 ± 7.3	21	49
Body height [m]	1.79 ± 8.7	1.59	1.95
Body mass [kg]	78.0 ± 8.3	58.5	94.8
BMI [kg·(m^2^)^−1^]	24.3 ± 2.9	18.1	30.9
Total training volume [h/week]	8.3 ± 4.3	2	20
Skitours [n/month]	9.4 ± 5.6	2	20
Elevation gain [m/tour]	1076 ± 224	750	1500
VO₂_max_ [ml·min⁻^1^ kg⁻^1^]	57.1 ± 5.8	48.1	65.8
HR_max_ [bpm]	189 ± 10	168	203
V_peak_ [km·h^-1^]	5.4 ± 0.6	4.6	6.4

SD, standard deviation; BMI, body mass index; VO₂_max_, maximal oxygen uptake; HR, heart rate; v_peak_, peak velocity at the end of the ramp protocol.

### Experimental design

The study consisted of two laboratory sessions for each participant. Both sessions were performed on a h/p/cosmos Saturn 300 cm × 125 cm treadmill (h/p/cosmos sports and medical GmbH, Germany) with participants being equipped at both sessions with a pair of Atomic Backland 78 Skies (169 cm), Atomic Backland Tour Binding (riser height: low, 0.0 cm; medium, 3.0 cm; high, 5.3 cm), and an Atomic Backland Sport Boot (Atomic Austria GmbH, Austria). Participants used standardized poles, which were changeable in length, and had an adjustable hand strap. Individual pole length was kept consistent for all testing sessions. There were at least 72 h and maximally 2 weeks between the first and the second session and the preparations as training, food, and time of test had to match. Furthermore, participants were asked not to train within 24 h prior to each session, and to abstain from caffeine and food for 5 h and 2 h prior, respectively, to their exercise session.

### Protocol—Performance test

During the first laboratory testing session, participants performed a specific performance test using touring skis on the treadmill to estimate their physiological fitness and to get used to the movement pattern on the treadmill. The test included a standardized warm-up of 5 minutes at 2.6 km h^-1^ and a gradient of 8%, with the riser height set at the medium position. After the warm-up, the measurement systems (ergospirometry system and heart rate sensor [HR]), which are described in detail later, were switched on and the incremental test protocol at a constant 16% elevation gradient was performed starting at 2.6 km h^-1^. After every 4-min interval, there was a break of 30 s to take a lactate (La) sample, before the speed increased by 0.4 km h^-1^. The step test was performed until a La level of ≥4 mmol L^-1^ was reached. After the last interval, the participants had a 3-min break, where the gradient was changed to 24% elevation and the ramp test protocol started. This protocol started at 2.6 km h^-1^ and the speed increased every minute by 0.4 km h^-1^ until participants reached their peak speed. The step test aimed to determine speed for the second session of testing, which was defined as the individual speed at a La value of 1.5 mmol L^-1^ (v_experiment_), which was 4.0 ± 0.5 km h^-1^ on average, with a minimum of 3.1 km h^-1^ and a maximum of 5.2 km h^-1^. The ramp protocol was conducted to obtain maximum oxygen uptake (VO_2max_), maximum HR and peak skimo velocity (Table 1).

### Protocol—Experimental test

The second testing session was the actual experimental session. In addition to the ergospirometry system, the HR sensor, and La analysis, the second test setting included surface electromyography (EMG), near-infrared spectroscopy (NIRS) and subjective scales (comfort scale and rating of perceived exertion). After a standardized warm-up of 5 minutes at v_experiment_ with the medium riser height setting and a gradient of 8%, the participant sat down on a chair, which was placed on the treadmill. The measuring systems were then switched on and 5 minutes of resting measurement followed. The test included three times 15 min walking in one gradient (8%, 16%, and 24%). Each 15 min block was split into three 5 min intervals, where the three riser height positions were applied randomly. The order of the gradients was the same for every participant, starting at 8%, followed by 16%, and ending at 24%. For data analysis, only data from the last minute of every 5-min interval was used. Between the 5 minutes intervals, the treadmill stopped for 1 minute to change the setting of the binding, take a blood sample, and to ask the participant for the ratings for the subjective scales. Between the 15 min blocks was a break of 2 minutes to additionally change the gradient.

### Measurements

For the first test, only physiological data were assessed, such as HR measured by a Wahoo Tickr HR belt (Wahoo Fitness, California, United States) and stored in the portable metabolic system (Cosmed K5, Cosmed, Rome Italy), which was set to breath-by-breath mode. The mobile gas analyzer was used to maximize freedom of movement while participating, even though the measurements took place indoors. The participants were breathing through a proper sized oronasal face mask, which was connected to a turbine flowmeter. The system was calibrated before every test in agreement with the manufacturer´s instructions. Fresh air circulation was given by open windows and an additional fan in front of the ski mountaineer to minimize cumulated exhaled air around the participant.

La samples were collected before the test, after every step, and one, three, and 5 minutes after volitional exhaustion. The blood samples of 20 μL were obtained from the earlobe and analyzed by an EKF-diagnostics Biosen C-line system (EKF-diagnostic GmbH, Germany).

Electromyography data were collected from the rectus femoris (RF), bicep femoris (BF), and medial gastrocnemius (GAS) of the left leg according to SENIAM recommendations (Stegeman and Hermens, 2007). Sensor sites were shaved and skin cleaned with isopropyl alcohol wipes. Sensors were then attached to the skin with two-sided tape, and surgical tape was then used to secure the sensors in place. Data were recorded on a portable data logger until processing (Trigno Personal Monitor, Delsys, Inc., Boston, MA). A 125 ms interval was used in the RMS calculation. The EMG signal was filtered with a second order Butterworth bandpass filter (20-500 Hz; Delsys, Inc., Boston, MA, United States). Sampling frequency was 1926 Hz. Due to some muscles being biarticular muscles and some affecting a triaxial joint, relative data are reported as a percent of total voltage range within each subject for better data presentation. The average voltage from each step cycle was divided by the voltage range to give a percent. Thirty step cycles from each interval were used in the calculation with the average being used in data analysis.

NIRS sensors (Idiag Moxy 5; Idiag AG, Switzerland) were placed on the right side of the body, matching the muscles used for EMG (RF, BF, and GAS). Data were stored on the internal memory, as well as on the portable metabolic system, which then allowed for data synchronization. The sensors were fixed with self-sticking pads and wrapped with a bandage to prevent falsified data due to light interference. Data were recorded at 0.5 Hz and is displayed as desaturation (DS) from baseline (BL). The measured tissue saturation index (TSI) was normalized intra-individually based on general recommendations (Perrey and Ferrari, 2018). The BL was taken from the last minute of the resting period right before the test started, and DS calculation follows the equation:

$$DS=\frac{TSI-BL}{BL}$$

To assess subjective perception, two scales were used (Grant et al., 1999): the Borg 6-20 scale (Robertson et al., 1998) to get the rating of perceived exertion (RPE), and a comfort scale (1-10), where 1 describes a very uncomfortable situation and 10 a very comfortable situation, which was already proven as valid and reliable in other context (Mündermann et al., 2002; Lee et al., 2013; Yusof et al., 2019). The comfort scale was applied separately for comfort of the lower body and comfort of the upper body.

Energy cost of linear and vertical displacement was calculated similar to Praz et al. (2016b), dividing the energy expenditure (J·s^-1^), which was obtained from the ergospirometry system, by the system mass (body mass +5 kg of gear and measurement systems) and the velocity (ms^-1^). For energy cost of linear displacement, velocity represents the walking velocity and for vertical energy cost, velocity represents vertical displacement velocity.

### Statistical analysis

For statistical calculations, SPSS, Version 27 (IBM Cooperation, United States) was used. For comparison of the different settings, a multifactorial ANOVA with repeated measurements was used to calculate the main effect of gradient and riser height of each dependent variable and their interaction. Whenever sphericity was not given (Mauchly Test, *p* < 0.05), Greenhouse-Geisser correction was applied for within-subjects effects. When a significant F value was found, Bonferroni’s test was used for pairwise comparisons. Whenever sphericity was not given (Mauchly Test *p* < 0.05), Greenhouse-Geisser correction was applied for within-subjects effects. Alpha level for significance was defined as < 0.05 and partial Eta squared (ηp^2^) is reported as effect size.

## Results

Global physiological response to gradient and riser height are shown in Table 2. The response of HR, VO_2_ and La measurements revealed an influence of gradient (*p* < 0.001), showing an increase in physiological response with an increase in gradient. But neither an effect of riser height (HR, *p* = 0.34; VO_2_, *p* = 0.26; La, *p* = 0.38) nor an interaction effect of gradient and riser height (HR, *p* = 0.3; VO_2_, *p* = 0.32; La, *p* = 0.73) was found.

**TABLE 2:** Global and local physiological response and subjective perception to gradient and riser height.

					ANOVA (*p*-value/ηp2)		
		Low RH	Med. RH	High RH	GR	RH	GR*RH
Heart rate [bpm]	8%	126 ± 15	127 ± 15	127 ± 13			
	16%	151 ± 15	151 ± 15	150 ± 15	**< 0.001/0.97**	0.338/0.06	0.299/0.07
	24%	176 ± 12	173 ± 14	173 ± 14			
VO₂ [ml·min⁻^1^ kg⁻^1^]	8%	32.1 ± 4.4	31.9 ± 3.7	32.5 ± 4.2			
	16%	41.7 ± 4.8	41.5 ± 4.5	41.4 ± 4.8	**< 0.001/0.96**	0.256/0.07	0.317/0.06
	24%	50.5 ± 6.1	51.4 ± 6.1	51.9 ± 6.2			
Lactate [mmol·L⁻^1^]	8%	0.9 ± 0.2	0.9 ± 0.2	0.9 ± 0.2			
	16%	1.5 ± 0.4	1.4 ± 0.3	1.4 ± 0.5	**< 0.001/0.79**	0.382/0.05	0.734/0.02
	24%	4.2 ± 1.5	4 ± 2.3	3.9 ± 1.8			
SmO_2_ GAS [DS]	8%	−42 ± 16	−30 ± 16	−24 ± 16			
	16%	−49 ± 16	−47 ± 17	−44 ± 17	**< 0.001/0.86**	**< 0.001/0.52**	**< 0.001/0.47**
	24%	−60 ± 15	−61 ± 14	−57 ± 17			
SmO_2_ RF [DS]	8%	−5 ± 13	−2 ± 13	−6 ± 15			
	16%	−8 ± 17	−8 ± 18	−9 ± 17	**< 0.001/0.71**	0.205/0.08	0.708/0.02
	24%	−26 ± 23	−25 ± 24	−27 ± 20			
SmO_2_ BF [DS]	8%	−13 ± 11	−7 ± 11	−9 ± 12			
	16%	−22 ± 14	−18 ± 14	−17 ± 14	**< 0.001/0.82**	**0.003/0.28**	0.422/0.05
	24%	−39 ± 16	−37 ± 17	−36 ± 13			
EMG GAS [% range]	8%	47.4 ± 6.1	45.2 ± 8.6	48.5 ± 5.7			
	16%	46.3 ± 5.2	48.8 ± 9.4	42.9 ± 8.3	0.645/0.02	0.235/0.08	**0.008/0.17**
	24%	45.7 ± 4.3	47.2 ± 5.7	44.9 ± 7.3			
EMG RF [% range]	8%	53 ± 6.7	52.5 ± 14.2	49.5 ± 14.6			
	16%	51.2 ± 6	52.8 ± 10.9	50.8 ± 17	0.865/< 0.01	0.884/< 0.01	0.812/0.02
	24%	50.5 ± 6.5	52.3 ± 21.4	54.3 ± 19.2			
EMG BF [% range]	8%	53.3 ± 8.3	46.1 ± 11.5	49.3 ± 15.1			
	16%	54.7 ± 7.4	50.8 ± 13.7	51.8 ± 15.1	0.355/0.06	0.069/0.14	**0.046/0.13**
	24%	53.8 ± 5.8	55.1 ± 13.7	46.5 ± 9.5			
RPE (Borg 6-20)	8%	8.5 ± 2.1	9.1 ± 1.8	10 ± 2.1			
	16%	13.4 ± 2.7	12.4 ± 1.6	12.6 ± 1.8	**< 0.001/0.9**	**0.012/0.22**	**0.001/0.23**
	24%	16.2 ± 2.2	15.5 ± 2.1	16.1 ± 2			
Comfort upper body (1-10)	8%	8.5 ± 1.4	7.8 ± 1.5	7.3 ± 2.2			
	16%	7.3 ± 1.4	7.6 ± 1.5	7.6 ± 1.5	**0.002/0.4**	0.516/0.04	**< 0.001/0.27**
	24%	5.4 ± 2.4	6.3 ± 2.6	6.2 ± 2.3			
Comfort lower body (1-10)	8%	8.6 ± 1.5	6.7 ± 2.1	4.3 ± 2.8			
	16%	6.1 ± 2.3	7.1 ± 1.8	6.6 ± 2.1	**0.016/0.23**	**< 0.001/0.42**	**< 0.001/0.57**
	24%	4.5 ± 2	6.4 ± 2.3	5.8 ± 2.1			

Mean ± standard deviation; RH, riser height; GR, gradient; GR x RH, interaction effect between gradient and riser height; med., medium; VO2, oxygen consumption; SmO2 [DS], muscle oxygen desaturation; EMG, electromyography; RPE, rating of perceived exertion.

**FIGURE 1:**
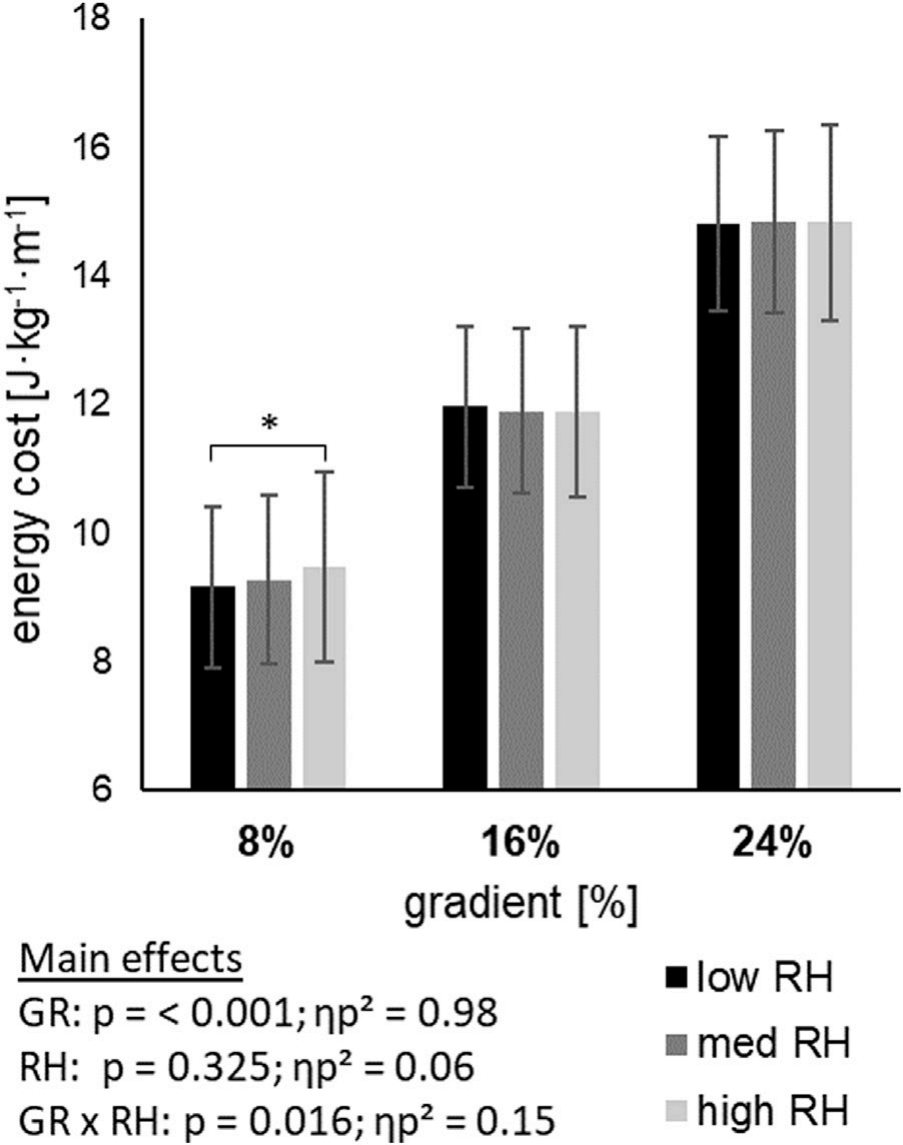
Energy cost of linear displacement across three gradients (GR), each including three riser heights (RH) mean ± standard deviation; GRx RH = interaction effect; * = pairwise comparisons within gradient; p=<0.05.

**FIGURE 2:**
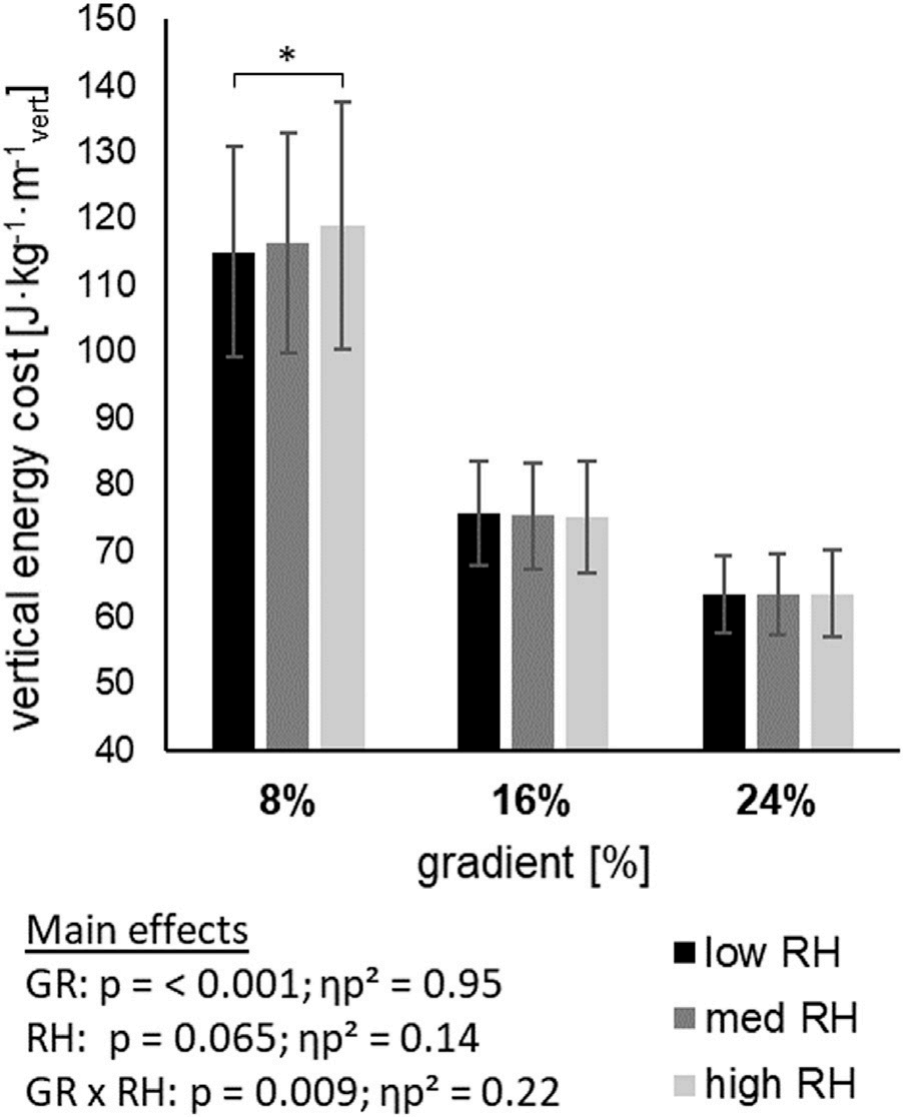
Energy cost of Verticle displacement across three gradients (GR), each including three riser heights (RH) mean ± standard deviation; GRx RH = interaction effect; * = pairwise comparisons within gradient; p=<0.05.

Energy cost of linear (Figure 1) and vertical (Figure 2) displacement demonstrated a main effect of gradient (*p* < 0.001), but not for riser height. With steeper gradients, energy cost of linear displacement increased, while energy cost of vertical displacement decreased. An interaction effect of gradient and riser height was found for linear (*p* = 0.016) and vertical (*p* = 0.009) energy cost. An increase of energy cost between riser heights was found within the 8% gradient, whilst no difference was found within 16%, and 24% gradient.

Muscle oxygen saturation (Table 2) of all three muscles (GAS, RF, BF) responded with an increase in desaturation to steeper gradients (*p* < 0.001). GAS (*p* < 0.001) and BF (*p* = 0.003) revealed a main effect of riser height, showing an increase in desaturation from high to low riser height. Additionally, GAS demonstrated an interaction effect of gradient and riser height (*p* < 0.001) with less difference in desaturation between the low and high riser height at 24%, compared to 8% gradient. RF (*p* = 0.71) and BF (*p* = 0.42) did not reveal an interaction effect.

EMG data (Table 2) show an interaction effect of gradient and riser height for GAS (*p* = 0.008) and BF (*p* = 0.046). However, neither an effect of gradient (GAS, *p* = 0.65; RF, *p* = 0.87; BF, *p* = 0.36) nor riser height (GAS, *p* = 0.24; RF, *p* = 0.88; BF, *p* = 0.07) was found for any of the three analysed muscles.

The RPE scale (Table 2) showed an effect of gradient (*p* < 0.001), with rising values from 8% to 24% gradient, and an effect of riser height (*p* = 0.012), with rising values from the low to the high riser height. The interaction effect of gradient and riser height (*p* = 0.001) is a consequence of different relations within the gradients. While the increase in RPE from low to high riser height is linear at 8% gradient, an asymmetric U-shape was found at 16% and 24% gradient. The comfort scale applied to the upper body showed a main effect of gradient (*p* < 0.001), but no effect of the applied riser height (*p* = 0.52) was observed. Comfort for the lower body revealed a main effect of the gradient (*p* = 0.016) and of the riser height (*p* < 0.001). Both, comfort of upper body and lower body revealed an interaction effect of gradient and riser height (*p* < 0.001). While comfort decreased from low to high riser height at 8% gradient, we found an increase from low to medium riser height at 16% and 24%, with no, or only minor changes compared to 24%.

## Discussion

The aim of this study was to investigate the influence of different riser heights and gradients in skimo on physiological and subjective variables.

Global physiological variables, HR, VO_2_, and La, showed an increase from 8% to 16% and 24% gradients, with a large effect size (0.97; 0.96; 0.79), respectively (Table 2). Due to the experimental setup of constant walking velocity, an increase in strain was to be expected, is evident in the data, and represented by increased energy cost. All three variables were not prone to detect changes related to the three available riser heights and therefore did not show a difference whether the low, medium, or high riser height was used. Energy cost of linear displacement also increased with the change in gradient from 8% to 24% (Figure 1). This finding is in line with work from Praz et al. (2016b). Although there was no effect of riser height, a significant interaction effect with a large effect size of gradient and riser height revealed that there is no difference with respect to riser height at 16% and 24%, but at 8% gradient. At the 8% gradient, the energy cost was greater for the high riser height compared to the low riser height, which means an advantage of the low riser height.

Therefore, the greater step length and higher range of motion for the ankle and knee joints with the low riser height (Lasshofer et al., 2022) were found simultaneously with a more efficient linear displacement, although local measurements like NIRS of GAS suggest increased use. Since the typical goal of ski mountaineering is to reach the top of a mountain, energy cost of vertical displacement is a decisive metric in skimo. Lowest vertical energy cost was found at 24%, followed by 16% and 8% gradient (Figure 2), which suggests choosing a steeper gradient, if possible, might save up to 50% of energy per vertical meter climbed when comparing 24%–8% gradient. These results are also supported by others (Praz et al., 2016a; Praz et al., 2016b; Lasshofer et al., 2022) who suggested steeper gradients being advantageous compared to flatter gradients. Although evidence is lacking, there must be a functional threshold in the natural environment of skimo, upon which steeper is not better anymore due to human capabilities, snow conditions or equipment capabilities, (e.g., skis starting to slip backward). Accounting for this study, riser height did not reveal a main effect on vertical energy cost, although a trend, suggesting the low riser height being advantageous, is evident (*p* = 0.065; ηp^2^ = 0.14). But a detailed look at vertical energy cost within the gradients based on an interaction effect of gradient and riser height (*p* = 0.009; ηp^2^ = 0.22) demonstrated the advantage of the low riser height at 8% gradient, while no difference at 16% and 24% was found. In contrast to these variables, subjective scales revealed not only an effect of gradient, but also of riser height. RPE on the one hand confirms the results of global physiological variables with a main effect of gradient (*p* < 0.001; ηp^2^ = 0.9), but on the other hand was also affected by the riser height (*p* = 0.012; ηp^2^ = 0.22). Consequently, global physiological measurements are not in line with perceived exertion. Whether these global measurements are not sensitive enough to detect changes, or muscular compensation mechanisms keep the overall strain constant remains unclear. Similar to energy cost, the low riser height was rated as the least strenuous option at the 8% gradient. Additionally, the medium riser height was found as least strenuous at the 16% and 24% gradients. The comfort scale was divided in upper body and lower body, with the aim to differentiate areas of influence. While upper and lower body comfort were affected by the gradient, with a reduction in comfort from 8% to 24%, only lower body comfort showed a main effect with large effect size (ηp^2^ = 0.42) of riser height. Specifically, the high riser height was clearly the least comfortable at 8%, which is perfectly in line with energy cost and RPE, followed by the medium and the low riser height. In contrast to the 8% result, at 16% and 24% gradient, the medium riser height was rated the most comfortable, followed by the high riser height, with the low riser height being the least comfortable at both the 16% and 24% gradients. This matches our hypothesis, that steeper gradients require a higher riser height, although we could not demonstrate the highest riser height being most comfortable at the steepest gradient.

Since the lower body kinematics are influenced by changes in riser height (Lasshofer et al., 2022), local muscular responses were investigated as well. Muscle oxygen desaturation represents the response of single muscles to exercise. While, once more, all three analyzed muscles confirm the greater strain or enhanced usage at steeper gradients (*p* < 0.001) by greater oxygen desaturation, GAS and BF were also prone to changes in riser height. It was shown, that a higher riser height functions as a supporter for the calf muscles and muscle oxygen desaturation in GAS is less with a higher riser height applied. There were similar results for the BF, with the low riser height resulting in greatest desaturation and strongest EMG signals (indicated by a strong trend in the effect of riser height (*p* = 0.069) and a large effect size (ηp^2^ = 0.14) over all gradients. Nevertheless, probably also due to the least general strain, at 8% gradient the low riser height, which showed greatest desaturation for GAS and BF, was rated as the most comfortable and perceived as the least strenuous one. EMG and NIRS signals of RF showed neither an effect of riser height, nor an interaction effect of riser height and gradient. This lines up perfectly with the fact of hip joint kinematics not being influenced by changes in RH (Lasshofer et al., 2022), since RF is a biarticular muscle also responsible for hip movement.

Though EMG signal and muscle oxygen desaturation of GAS and BF suggest preferring the high riser height at 24%, RPE and comfort scale emphasize to apply the medium riser height. In other sports, NIRS signal was shown to be affected by cadence, where greater oxygen saturation levels were generally found with higher cadence (Zorgati et al., 2013; Steimers et al., 2016). In our specific case of skimo, the trend of less desaturation with higher cadence (Lasshofer et al., 2022) is also evident. But with respect to the intervention of not manipulating cadence, but cadence being adapted to changes in riser height and therefore manipulating whole body kinematics, we cannot assume cadence as the only reason for changes in muscle desaturation. Because at the same time, we also find a reduction in ankle joint range of motion, which can also be associated with less desaturation. Because global physiology was not affected by riser height, we suggest applying the most comfortable and perceived least exhausting riser height, since evident differences in local muscular strain were compensated elsewhere. This most comfortable choice can be supported at 8% gradient by energy cost, and at steeper gradients with EMG and NIRS analysis.

## Limitations

This study was conducted in a laboratory setting. This allowed for strict standardization of testing and provided consistency. Although regular skimo equipment was used, walking on the treadmill might be somewhat different to walking on snow and the maximum gradient was limited to 24%. Other authors reported similar results comparing on snow and treadmill skimo for the available gradients (Tosi et al., 2010; Praz et al., 2016a; b). Unfortunately, we were not able to extrapolate the results to steeper gradients (based on the maximal possible gradient of the treadmill), which can be found in outdoor skimo We could only hypothesize the high riser height gaining more relevance in steeper terrain, but this must be tested in another study.

We decided to apply a constant speed over all three tested gradients, which was tested and defined during the first testing session. Pilot testing prior to the study showed that participants were not able to walk at self-selected speed on the treadmill, especially applying an uncomfortable riser height.

## Conclusion and practical application

In conclusion, even though global physiological parameters were similar between riser heights, local measurements of NIRS and EMG, perceived exertion and comfort can differ between the situations. Supported by energy cost, we demonstrated, a benefit of the low riser height at 8% gradient. While in general, at 16% the medium riser height showed benefits, and at 24% the medium and the high riser height outperformed the low riser height clearly with varying strengths. Based on the parameters and gradients analyzed in this study, it can be concluded that only the low and medium riser heights provided a benefit to the skiers, supported by subjective scales, local measurements and energy expenditure.

## Data Availability

The raw data supporting the conclusion of this article will be made available by the authors, without undue reservation.
